# Pembrolizumab-Associated Secondary Adrenal Insufficiency Due to Isolated Adrenocorticotropic Hormone (ACTH) Deficiency Presenting as Recurrent Multisystem Decompensation in Triple-Negative Breast Cancer

**DOI:** 10.7759/cureus.118005

**Published:** 2026-10-16

**Authors:** Niyas Khalid Ottu Para, Asiya Pathan, Mohamed A Babiker, Seema Rab

**Affiliations:** 1 Internal Medicine, Burjeel Hospital, Abu Dhabi, ARE; 2 Internal Medicine, Burjeel Holdings, Abu Dhabi, ARE; 3 Clinical Oncology, National Cancer Institute, Wad-Madani, SDN

**Keywords:** adrenal insuficiency, chemotherapy response, immune-checkpoint inhibitors, pembrolizumab side effect, triple-negative breast cancer

## Abstract

Immune checkpoint inhibitors have transformed the treatment of high-risk triple-negative breast cancer (TNBC), yet endocrine immune-related adverse events remain challenging to recognize because their manifestations frequently overlap with infection, chemotherapy toxicity, malignancy progression, and cardiovascular disease. Among these toxicities, isolated adrenocorticotropic hormone (ACTH) deficiency is uncommon and may evolve insidiously before presenting as clinically significant secondary adrenal insufficiency.

We report a 40-year-old woman with BRCA-positive triple-negative invasive ductal carcinoma receiving neoadjuvant carboplatin, paclitaxel, and pembrolizumab who experienced recurrent hospitalizations over several months with abdominal pain, nausea, constitutional decline, inflammatory episodes, hypotension, atrial fibrillation with rapid ventricular response, severe electrolyte disturbances, and repeated sepsis-like decompensations. Interpretation was further complicated by a history of mechanical mitral valve replacement requiring chronic anticoagulation.

Longitudinal reassessment revealed recurrent metabolic instability characterized by severe hypomagnesemia, hypercalcemia with suppressed parathyroid hormone (PTH), ACTH suppression (0.3 pmol/L), severe hypocortisolemia (1.8 µg/L), and inadequate Synacthen response despite normal pituitary magnetic resonance imaging (MRI). Intravenous hydrocortisone resulted in rapid clinical recovery, followed by discharge on physiologic glucocorticoid replacement.

This case highlights pembrolizumab-associated MRI-negative central adrenal insufficiency presenting within a prolonged pattern of recurrent multisystem deterioration. Severe cortisol deficiency may impair physiologic stress tolerance and complicate the interpretation of concurrent oncologic, cardiovascular, inflammatory, and metabolic abnormalities. In patients receiving programmed death-1 (PD-1) inhibitors, recurrent unexplained hypotension, constitutional decline, or sepsis-like presentations should prompt early assessment of the hypothalamic-pituitary-adrenal axis, even when pituitary imaging is normal.

## Introduction

Triple-negative breast cancer (TNBC) is a biologically aggressive subtype of breast malignancy characterized by the absence of estrogen receptor, progesterone receptor, and human epidermal growth factor receptor-2 expression. Compared with hormone receptor-positive breast cancers, TNBC demonstrates greater genomic instability, increased proliferative activity, enhanced immune infiltration, and higher tumor immunogenicity. BRCA-associated TNBC may further amplify these features through homologous recombination deficiency and increased neoantigen generation, providing a biologic rationale for immune-based therapeutic strategies [1,2].

Pembrolizumab is a monoclonal antibody directed against programmed death-1 (PD-1), an inhibitory immune checkpoint that regulates peripheral immune tolerance. By disrupting PD-1 signaling, pembrolizumab restores cytotoxic T-cell activity and enhances antitumor immunity. The KEYNOTE-522 trial established pembrolizumab-containing neoadjuvant therapy as a standard treatment strategy for high-risk early-stage TNBC after demonstrating improvements in pathological complete response, event-free survival, and overall survival [3]. Therapeutic enhancement of immune activity, however, is accompanied by loss of immune tolerance, permitting autoreactive responses against normal tissues and resulting in immune-related adverse events affecting multiple organ systems, particularly endocrine organs [4-6].

Among endocrine immune-related adverse events, isolated adrenocorticotropic hormone (ACTH) deficiency and secondary adrenal insufficiency are uncommon but clinically important complications. In contrast to cytotoxic T-lymphocyte antigen-4 (CTLA-4) inhibitor-associated hypophysitis, which frequently presents with pituitary enlargement and multiple hormonal deficiencies, PD-1 inhibitor-associated pituitary dysfunction more commonly manifests as isolated corticotroph failure with minimal or absent radiologic abnormalities [7-10]. Consequently, clinically significant adrenal insufficiency may develop despite entirely normal pituitary imaging.

Recognition is often delayed because symptoms overlap with infection, chemotherapy toxicity, malignancy progression, cachexia, cardiovascular instability, and critical illness. As a result, evolving adrenal insufficiency may remain unrecognized until recurrent hemodynamic deterioration or adrenal crisis-like physiology emerges [11-13].

We report a case of pembrolizumab-associated magnetic resonance imaging (MRI)-negative secondary adrenal insufficiency due to isolated ACTH deficiency in a patient with BRCA-positive TNBC whose endocrine dysfunction evolved progressively across multiple admissions and ultimately presented as recurrent metabolic, inflammatory, and cardiovascular decompensation. The case highlights the importance of longitudinal diagnostic synthesis in patients receiving immune checkpoint inhibitors and illustrates how endocrine toxicity may manifest as multisystem physiologic destabilization rather than classical endocrine disease.

## Case presentation

A 40-year-old woman with recently diagnosed right-sided triple-negative invasive ductal carcinoma developed recurrent episodes of constitutional decline, inflammatory deterioration, metabolic instability, and cardiovascular decompensation. She developed progressively worsening nonspecific symptoms including persistent vomiting, nausea, abdominal pain, dizziness, fatigue, generalized weakness, reduced exercise tolerance, diffuse body pain, and neuropathic symptoms affecting the extremities.

Earlier oncologic evaluation had demonstrated invasive carcinoma of no special type (ductal carcinoma), grade 3, with negative estrogen receptor, progesterone receptor, and HER2 expression. The proliferative index (Ki-67) was approximately 90%, and germline testing confirmed BRCA positivity. Tumor board evaluation documented a metabolically active right breast lesion without convincing evidence of distant metastatic disease. Baseline staging investigations demonstrated cardiomegaly related to prior mechanical mitral valve replacement and atrial dilation but no disseminated malignancy or adrenal involvement.

The patient had substantial baseline cardiovascular complexity. Her medical history included mechanical mitral valve replacement requiring long-term anticoagulation, chronic atrial fibrillation, and recurrent anticoagulation-related management challenges. Because of elevated cardiac risk, anthracycline-based chemotherapy was considered unfavorable, and neoadjuvant treatment with carboplatin, paclitaxel, and pembrolizumab was initiated in October 2025. Glucocorticoid exposure during chemotherapy was limited to intermittent short courses of dexamethasone, typically 4 mg twice daily for three days. The patient had no history of chronic systemic glucocorticoid therapy. The final dexamethasone exposure occurred approximately four months before endocrine assessment, with no further systemic glucocorticoid administration during the intervening period. This prolonged steroid-free interval was considered important when evaluating exogenous glucocorticoid-induced hypothalamic-pituitary-adrenal (HPA) axis suppression as an alternative diagnosis.

Although treatment was initially tolerated, constitutional and inflammatory symptoms began emerging during subsequent treatment cycles and progressed over the ensuing months. The early clinical course was not marked by any clearly recognizable endocrine syndrome. Instead, the patient developed progressively worsening nonspecific symptoms that evolved across multiple encounters. Initial manifestations included diffuse body pain, generalized weakness, fatigue, reduced exercise tolerance, intermittent nausea, episodic abdominal discomfort, neuropathic “electrical sensation” symptoms involving the extremities, and progressive reduction in perceived physiologic reserve. These symptoms fluctuated in severity and were initially interpreted within the broader context of malignancy, chemotherapy-related toxicity, systemic inflammation, and physical deconditioning.

By late 2025 and early 2026, repeated emergency visits and admissions became increasingly frequent. Several presentations were characterized by abdominal pain, nausea, vomiting, hypotension, constitutional decline, tachyarrhythmia, and inflammatory marker elevation. During these episodes, infectious etiologies became dominant diagnostic considerations because of fever, hemodynamic instability, elevated inflammatory markers, malignancy-associated immunologic vulnerability, and prosthetic valve status. Port-related infection, prosthetic valve endocarditis, systemic infection, and immunocompromised host sepsis were repeatedly considered. Broad-spectrum antimicrobial escalation, intensive monitoring, multidisciplinary evaluations, and recurrent ICU-level admissions for septic or distributive physiology followed several admissions. A detailed chronology of oncologic treatment, admissions, and endocrine evolution is summarized in Table 1.

**Table 1:** Chronological evolution leading to diagnosis of pembrolizumab-associated secondary adrenal insufficiency. TNBC: triple-negative breast cancer; PD-1: programmed death-1; MRI: magnetic resonance imaging; ACTH: adrenocorticotropic hormone

Period	Clinical course	Key findings	Diagnostic significance
July-August 2025	Initial workup and multidisciplinary tumor board review for BRCA-positive grade 3 TNBC	High-risk TNBC; no distant metastatic disease identified	Established indication for neoadjuvant pembrolizumab-containing therapy
October 2025	Initiation of carboplatin, paclitaxel, and pembrolizumab	Treatment initially well tolerated	Beginning of PD-1 inhibitor exposure
November-December 2025	Completion of early treatment cycles with intermittent infusion reactions and evolving constitutional symptoms	Fatigue, nausea, declining exercise tolerance; brief chemotherapy-associated dexamethasone courses (4 mg twice daily for three days); no chronic glucocorticoid treatment	Early manifestations potentially masked by treatment-related symptoms and corticosteroid administration
January 2026	Radiologic reassessment and surgical planning	Near-complete radiologic tumor response; no evidence of disease progression	Malignancy progression became less likely as an explanation for subsequent clinical deterioration
February-March 2026	Progressive constitutional decline with recurrent healthcare encounters	Generalized pain, neuropathic symptoms, weakness, reduced functional capacity, recurrent emergency visits	Evolving endocrine dysfunction remained unrecognized because symptoms were nonspecific
March 2026	Major hospitalization with ICU-level concern	Hypotension, atrial fibrillation with rapid ventricular response, inflammatory marker elevation, severe hypomagnesemia and hypokalemia	Initially managed as sepsis, cardiovascular instability, or treatment-related toxicity
April 2026	Recurrent admissions and extensive investigations	Persistent metabolic and inflammatory abnormalities without a convincing infectious source; gastric and lymph node biopsies negative for malignant or infectious pathology	Pattern of recurrent physiologic decompensation became increasingly apparent
Early May 2026	Integrative endocrine review, dynamic endocrine testing, pituitary MRI, initiation of glucocorticoid therapy	Marked ACTH suppression, severe hypocortisolemia, failed Synacthen test, normal pituitary MRI. Rapid improvement in constitutional symptoms following initiation of hydrocortisone	Recognition and confirmation of MRI-negative secondary adrenal insufficiency due to isolated ACTH deficiency

Retrospective longitudinal review identified recurrent metabolic and inflammatory abnormalities accompanying the patient's repeated hospitalizations. Importantly, glucose measurements during episodes of illness before glucocorticoid replacement documented values as low as approximately 3.4-3.5 mmol/L, demonstrating an intermittent low-glucose tendency independently of the HbA1c result. Additional abnormalities included recurrent severe hypomagnesemia (0.385 mmol/L), hypokalemia (3.2 mmol/L), intermittent bicarbonate reduction, fluctuating renal function, hypercalcemia (2.65 mmol/L) with suppressed parathyroid hormone (PTH) (0.37 pmol/L), and elevated inflammatory markers. Although these findings were not individually specific to adrenal insufficiency, their recurrence alongside hypotension and constitutional deterioration prompted consideration of impaired endocrine stress adaptation.

A prominent biochemical feature throughout the illness trajectory was recurrent severe hypomagnesemia accompanied by potassium and phosphate abnormalities. Episodes of atrial fibrillation with rapid ventricular response frequently coincided with electrolyte collapse and inflammatory deterioration, raising concern for cardiovascular destabilization secondary to systemic illness, infection, chemotherapy toxicity, or worsening cardiac disease. Serum creatinine, typically normal at baseline, intermittently increased during physiologic decompensation before returning toward baseline following stabilization.

Several admissions additionally demonstrated recurrent disturbances in calcium homeostasis. During one representative evaluation, corrected calcium measured 2.64 mmol/L with elevated ionized calcium of 1.57 mmol/L, while PTH was markedly suppressed at 0.37 pmol/L. Amylase and lipase remained at the lower limits of normal. Because of recurrent abdominal pain, nausea, vomiting, calcium abnormalities, and low-normal pancreatic enzymes, structural pancreatic disease, MEN-spectrum pathology, occult malignancy progression, metastatic disease, and endocrine neoplasia entered the differential diagnosis.

An admission in March 2026 represented a major turning point. The patient developed worsening inflammatory physiology with severe hypomagnesemia, hypokalemia, recurrent hypotension, atrial fibrillation with rapid ventricular response, constitutional decline, and ICU-level concern for septic or distributive physiology. Despite aggressive supportive care, repeated infectious evaluation, and multidisciplinary review, no convincing unifying explanation emerged.

Given the recurrent multisystem deterioration and diagnostic complexity, several competing explanations were systematically considered during the patient’s clinical course. Given the patient's mechanical mitral valve and recurrent febrile and sepsis-like presentations, bloodstream infection and prosthetic-valve endocarditis were repeatedly investigated. Serial aerobic and anaerobic blood cultures and additional microbiological specimens were predominantly negative, without evidence of persistent or reproducible bacteremia. A single body-fluid culture yielded scant growth of *Staphylococcus epidermidis*, which was clinically considered a contaminant. Transthoracic echocardiography performed during the period of recurrent deterioration in April 2026 demonstrated a functioning mechanical mitral prosthesis without leak, vegetation, or thrombus, with preserved left ventricular systolic function (estimated ejection fraction 58%-63%). The report specifically documented no echocardiographic evidence of infective endocarditis. Although these investigations could not categorically exclude every infectious process, no convincing persistent microbiological or structural infectious source was identified to explain the recurrent pattern of deterioration. Metastatic disease and occult endocrine infiltration were also considered because of aggressive TNBC biology, constitutional decline, hypercalcemia, and repeated admissions; however, positron emission tomography (PET) and cross-sectional imaging demonstrated no adrenal metastases, disseminated malignancy, or pituitary lesions.

Structural pancreatic pathology and MEN-spectrum disorders were entertained because of recurrent abdominal pain, nausea, vomiting, hypercalcemia, suppressed PTH, and low-normal pancreatic enzymes, but abdominal imaging remained unrevealing, and the biochemical profile lacked evidence of hormonally active endocrine neoplasia. The markedly suppressed ACTH accompanying severe hypocortisolemia supported central rather than primary adrenal insufficiency. Synacthen testing confirmed impaired adrenal reserve, while adrenal imaging demonstrated no structural abnormality. Renin and aldosterone were not measured. Antiphospholipid syndrome and thrombo-inflammatory disease were considered because of thrombotic complexity and anticoagulation history, although lupus anticoagulant and anticardiolipin antibodies were negative. Diabetic ketoacidosis and pancreatic endocrine dysfunction were also considered because of metabolic disturbance and abdominal symptoms but were not supported by persistent hyperglycemia, glycemic indices, or pancreatic imaging findings.

The recurrent coexistence of abdominal symptoms, low glucose tendency, bicarbonate reduction, hypercalcemia with suppressed PTH, inflammatory deterioration, constitutional decline, recurrent hypotension, and unexplained hemodynamic instability ultimately prompted broader endocrine consideration during admission in May 2026. The reproducibility of these abnormalities across multiple admissions, together with the absence of a convincing unifying infectious or structural explanation, shifted diagnostic consideration toward checkpoint inhibitor-associated endocrine dysfunction.

Endocrine evaluation was performed before initiation of hydrocortisone replacement, approximately four months after the last systemic glucocorticoid exposure. ACTH was markedly suppressed at 0.3 pmol/L (reference interval 1.6-13.9 pmol/L), accompanied by severe hypocortisolemia. A standard-dose Synacthen stimulation test was performed. Baseline blood was collected at 08:04, followed by intramuscular administration of 250 µg Synacthen at 08:10. Subsequent cortisol samples were obtained at 08:40 and 09:12, corresponding to 30 and 62 minutes after administration. Serum cortisol was measured using the Roche Elecsys Cortisol II competitive electrochemiluminescence immunoassay (ECLIA) on the cobas e 801 analyzer (Roche Diagnostics, Basel, Switzerland). Cortisol concentrations were 5, 91, and 116 nmol/L (equivalent to 1.8, 33, and 42 µg/L), respectively, demonstrating a markedly inadequate stimulated response. Serum albumin on the testing day was 29.6 g/L. Although hypoalbuminemia may influence total cortisol measurements, the markedly impaired stimulated response, together with suppressed ACTH, strongly supported central adrenal insufficiency. The clinical and treatment chronology favored pembrolizumab-associated corticotroph dysfunction.

Additional pituitary evaluation demonstrated reduced insulin-like growth factor-1 (IGF-1) at 5.440 nmol/L (reference interval 11.970-29.740 nmol/L) and mildly elevated prolactin at 34.2 µg/L. Reduced IGF-1 was interpreted cautiously in the setting of systemic illness and possible nutritional or catabolic influences, without establishing growth hormone deficiency. Mild hyperprolactinemia was nonspecific. Free thyroid hormone concentrations were within reference intervals, while luteinizing hormone (LH) and follicle-stimulating hormone (FSH) were not suppressed; however, gonadal-axis interpretation was limited by the absence of complete contemporaneous reproductive hormonal assessment. ACTH deficiency therefore represented the unequivocally demonstrated and clinically predominant pituitary hormonal deficit. HbA1c was 4.0%; separate glucose measurements during preceding illness episodes documented values of approximately 3.4-3.5 mmol/L. Lupus anticoagulant and anticardiolipin antibodies were negative.

Pituitary MRI with contrast demonstrated a structurally normal pituitary gland without enlargement, stalk thickening, abnormal enhancement, suprasellar lesion, or infiltrative abnormality (Figure 1). Computed tomography (CT) (Figure 2) and PET similarly failed to demonstrate adrenal metastases or structural adrenal pathology. Key endocrine, biochemical, and metabolic findings are summarized in Table 2.

**Figure 1:**
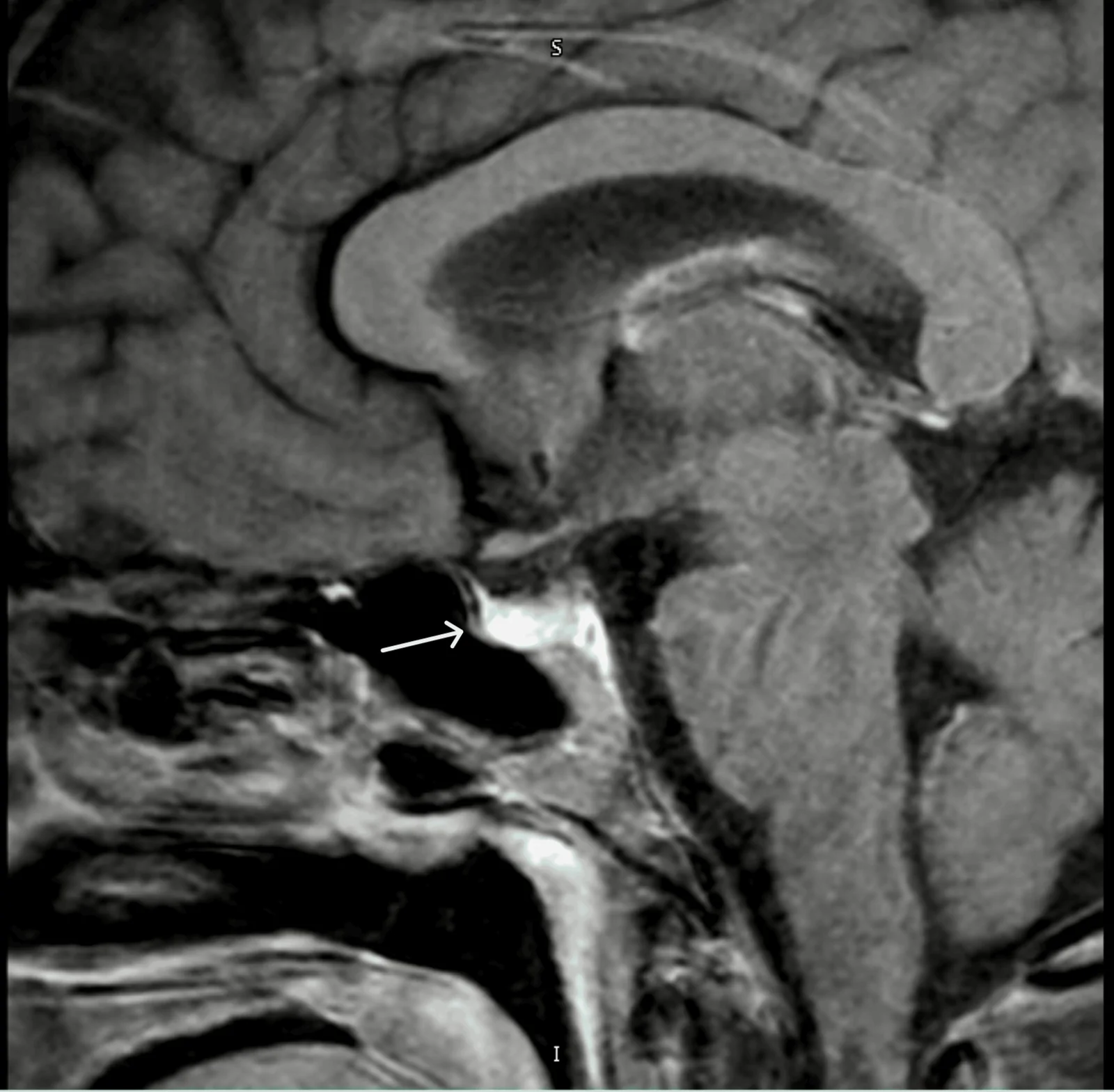
Post-contrast sagittal T1-weighted magnetic resonance image of the pituitary region. Gadolinium-enhanced, fat-suppressed T1-weighted imaging demonstrates no obvious pituitary enlargement or focal sellar mass. The pituitary gland is indicated by the arrow and demonstrates normal size and morphology, without enlargement, stalk thickening, abnormal enhancement, suprasellar lesion, or infiltrative abnormality.

**Figure 2:**
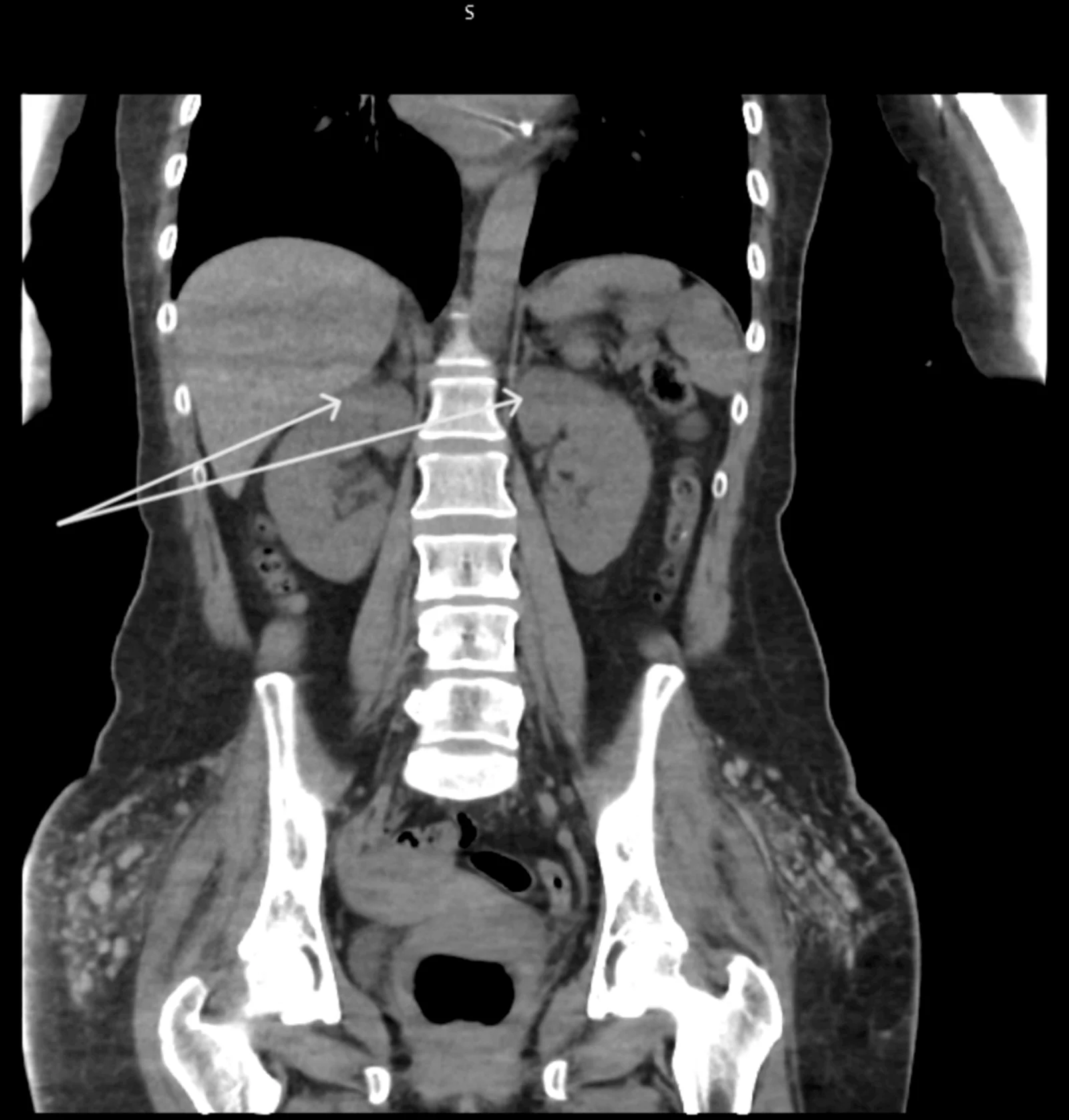
Contrast-enhanced computed tomography (CT) of the abdomen and pelvis. Right and left adrenal glands are indicated by arrows. Both adrenal glands demonstrate normal morphology without enlargement, focal lesion, infiltration, or evidence of metastatic involvement.

**Table 2:** Key endocrine, biochemical, and metabolic findings. ACTH: adrenocorticotropic hormone; IGF-1: insulin-like growth factor-1; LH: luteinizing hormone; FSH: follicle-stimulating hormone; FT3: free triiodothyronine; FT4: free thyroxine; TSH: thyroid-stimulating hormone; PTH: parathyroid hormone; MRI: magnetic resonance imaging; CT: computed tomography

Parameter	Result	Reference range	Interpretation
ACTH	0.3 pmol/L	1.6-13.9 pmol/L	Profoundly suppressed
Baseline cortisol	1.8 µg/L	23.2-194.3 µg/L	Severe hypocortisolemia
30-minute Synacthen cortisol	33.0 µg/L	>155 µg/L	Inadequate adrenal response
60-minute Synacthen cortisol	42.0 µg/L	>155 µg/L	Inadequate adrenal response
IGF-1	5.44 nmol/L	11.97-29.74 nmol/L	Reduced
Prolactin	34.2 µg/L	4.8-23.3 µg/L	Mildly elevated
LH	18.8 IU/L	2.4-95.6 IU/L	Preserved gonadotroph axis
FSH	43.4 IU/L	16.7-113.6 IU/L	Preserved gonadotroph axis
FT3	3.36 pmol/L	3.10-6.80 pmol/L	Normal
FT4	17.8 pmol/L	12.0-22.0 pmol/L	Normal
TSH	0.502 mIU/L	0.27-4.20 mIU/L	Normal
HbA1c	4.0%	4.0%-5.6%	Low-normal; consistent with recurrent hypoglycemia tendency
Corrected calcium	2.65 mmol/L	2.10-2.55 mmol/L	Elevated
Ionized calcium	1.57 mmol/L	1.12-1.32 mmol/L	Elevated
PTH	0.37 pmol/L	1.60-6.90 pmol/L	Suppressed
Magnesium	0.385 mmol/L	0.66-1.07 mmol/L	Severe hypomagnesemia
Potassium	3.2 mmol/L	3.5-5.1 mmol/L	Hypokalemia
Bicarbonate	18 mmol/L	22-29 mmol/L	Metabolic acidosis
Pituitary MRI	Normal pituitary gland	Normal study	MRI-negative hypophysitis phenotype
CT abdomen/pelvis	Normal adrenal glands	Normal study	No structural adrenal pathology

Taken together, pembrolizumab exposure, marked ACTH suppression, severe hypocortisolemia, impaired stimulated cortisol response, normal pituitary imaging, and the absence of a convincing structural alternative supported the clinical diagnosis of pembrolizumab-associated central adrenal insufficiency due to ACTH deficiency. The patient was initiated on intravenous hydrocortisone at 100 mg twice daily. Clinical recovery was rapid and clinically striking. Hemodynamic stability improved, nausea and constitutional symptoms lessened, inflammatory and metabolic deterioration stabilized, and the recurrent cycle of physiologic decline appeared to abruptly reverse following glucocorticoid replacement. The clinical response was sufficiently marked to strongly support clinically meaningful adrenal insufficiency rather than incidental biochemical abnormality. She was subsequently transitioned to oral hydrocortisone replacement consisting of 20 mg in the morning and 10 mg in the evening and discharged with endocrine follow-up. In retrospect, the patient’s illness represented a progressive endocrine-metabolic syndrome unfolding across fragmented healthcare encounters, in which recurrent inflammatory crises, metabolic instability, cardiovascular deterioration, electrolyte collapse, and repeated sepsis-like presentations reflected evolving failure of physiologic stress adaptation rather than isolated unrelated clinical events.

Follow-up and outcome

Following initiation of intravenous hydrocortisone and transition to oral replacement therapy at 30 mg/day (20 mg in the morning and 10 mg later in the day), the patient demonstrated sustained clinical and functional improvement. Dizziness, fatigue, generalized weakness, and exercise intolerance improved substantially. Whereas she had previously required wheelchair assistance during several outpatient visits, she subsequently attended follow-up independently and ambulatory.

Importantly, no further comparable sepsis-like episodes, unexplained hypotensive decompensations, or adrenal crisis-like events were reported following initiation of glucocorticoid replacement. As clinical stability was maintained, oral hydrocortisone was subsequently reduced from 30 to 20 mg/day.

The patient later underwent planned definitive breast surgery with perioperative stress-dose hydrocortisone coverage. The procedure and postoperative course were uncomplicated, without recurrence of hemodynamic instability, metabolic deterioration, or adrenal crisis. She successfully transitioned back to oral replacement therapy and remained clinically stable at the most recent follow-up. Continued endocrine follow-up was planned for assessment of replacement requirements, clinical stability, and appropriate stress-dose management during intercurrent illness or future procedures.

Patient perspective

At follow-up after initiation of hydrocortisone replacement therapy, the patient reported a substantial improvement in overall well-being. Symptoms of dizziness, fatigue, weakness, and exercise intolerance had markedly improved compared with previous admissions. The patient, who had frequently required wheelchair assistance during earlier clinic visits because of generalized weakness and functional decline, was able to attend follow-up ambulatory and reported a significant improvement in daily functioning. She expressed satisfaction with the diagnosis and treatment plan and described a noticeable improvement in quality of life following glucocorticoid replacement.

## Discussion

This case illustrates how immune checkpoint inhibitor-associated isolated ACTH deficiency may evolve as a syndrome of progressive physiologic stress failure rather than a recognizable endocrine disorder. Over several months, the patient experienced recurrent inflammatory crises, cardiovascular instability, electrolyte collapse, metabolic dysfunction, abdominal symptomatology, and repeated ICU-level decompensations that were initially interpreted through infectious, oncologic, cardiovascular, and treatment-related frameworks. Rather than presenting with classical features of adrenal insufficiency, endocrine dysfunction emerged as a pattern of recurrent multisystem physiologic deterioration occurring across fragmented healthcare encounters. The significance of this case lies not solely in the diagnosis of pembrolizumab-associated isolated ACTH deficiency, but in the demonstration of how evolving corticotroph failure can masquerade as recurrent sepsis-like physiology, and the repeated clinical deterioration suggested reduced tolerance to concurrent physiological stressors in a patient subsequently found to have severe central adrenal insufficiency.

The oncologic context is central to understanding the therapeutic exposure and the diagnostic complexity. BRCA-positive TNBC is a biologically aggressive and immunologically active malignancy characterized by high proliferative activity, genomic instability, increased neoantigen burden, and enrichment of tumor-infiltrating lymphocytes. These biologic characteristics have supported integration of immune checkpoint inhibition into contemporary treatment paradigms, particularly following the KEYNOTE-522 program demonstrating improvements in pathologic complete response, event-free survival, and overall survival with pembrolizumab-containing neoadjuvant therapy [1-3].

Pembrolizumab is a humanized monoclonal antibody directed against PD-1, an inhibitory immune checkpoint receptor expressed predominantly on activated T lymphocytes. Under physiologic conditions, PD-1 signaling functions as an essential regulator of peripheral immune tolerance through interaction with programmed death ligand-1 (PD-L1) and PD-L2, attenuating T-cell activation, limiting inflammatory injury, and suppressing autoreactivity. Tumor cells exploit this pathway through PD-L1 expression, inducing T-cell exhaustion and escaping immune surveillance. Pharmacologic interruption of PD-1 signaling restores antitumor cytotoxicity through expansion of activated T-cell populations, interferon-γ-mediated immune signaling, and reactivation of immune surveillance pathways. However, therapeutic restoration of immune activity simultaneously weakens mechanisms of peripheral tolerance and may permit autoreactive immune responses directed against normal tissues, including endocrine organs. Loss of immune tolerance may permit autoreactive CD8+ T-cell activation, interferon-mediated inflammatory signaling, and selective endocrine tissue injury without necessarily producing extensive structural inflammation detectable radiologically. Endocrine immune-related adverse events therefore represent a mechanistically coherent consequence of immune restoration rather than isolated idiosyncratic toxicity [4-8].

The molecular and clinicoradiologic behavior of PD-1 inhibitor-associated pituitary dysfunction differs substantially from classical CTLA-4 inhibitor hypophysitis. CTLA-4 inhibitor toxicity, particularly with ipilimumab, frequently produces diffuse pituitary enlargement, stalk thickening, headache, mass effect symptoms, and multiple anterior pituitary hormonal deficiencies, partly mediated through complement activation and immune targeting of ectopic CTLA-4 expression within pituitary tissue [9,10]. In contrast, PD-1 inhibitor-associated disease more commonly manifests as selective corticotroph dysfunction, often with absent or minimal radiologic abnormalities. PD-1 inhibitor-associated pituitary dysfunction frequently presents as ACTH-predominant deficiency without the radiological changes classically associated with CTLA-4 inhibitor-related hypophysitis [11-14]. The underlying immune mechanisms remain incompletely established, and direct evidence of corticotroph injury was not available in this patient.

The present case exemplifies this increasingly recognized MRI-negative phenotype. Despite marked ACTH suppression, severe hypocortisolemia, and clinically meaningful adrenal insufficiency, pituitary MRI demonstrated no enlargement, stalk thickening, abnormal enhancement, or infiltrative pathology. This imaging pattern is mechanistically aligned with contemporary descriptions of pembrolizumab-associated isolated ACTH deficiency and highlights an important contributor to delayed recognition in clinical practice [11-15].

The endocrine profile strongly supported secondary adrenal insufficiency rather than primary adrenal destruction. ACTH was profoundly suppressed at 0.3 pmol/L while baseline cortisol measured only 1.8 µg/L. Synacthen stimulation demonstrated a markedly suboptimal response, with cortisol increasing to only 33 µg/L at 30 minutes and 42 µg/L at 60 minutes, remaining substantially below the expected threshold for adequate adrenal reserve. This pattern is consistent with evolving central corticotroph dysfunction causing impaired HPA axis signaling. Absence of adrenal lesions on PET-CT and cross-sectional imaging further reduced the likelihood of metastatic adrenal infiltration.

The expected relative preservation of aldosterone secretion in central adrenal insufficiency may contribute to diagnostic difficulty, although mineralocorticoid function was not directly measured in this patient. In secondary adrenal insufficiency, aldosterone secretion remains predominantly regulated through the renin-angiotensin-aldosterone system rather than ACTH. Consequently, patients may not develop the classical biochemical phenotype of primary adrenal failure, including marked hyperkalemia or salt-wasting physiology. Absence of overt mineralocorticoid dysfunction may therefore provide false reassurance and delay recognition, particularly when inflammatory deterioration and cardiovascular instability dominate the clinical picture.

A central physiologic concept emerging from this case is progressive failure of stress adaptation. Cortisol functions not merely as a metabolic hormone but as a key regulator of systemic resilience during inflammatory, infectious, cardiovascular, and metabolic stress states. Adequate glucocorticoid signaling maintains vascular tone, endothelial integrity, catecholamine responsiveness, gluconeogenesis, mitochondrial energy adaptation, inflammatory regulation, and metabolic flexibility during physiologic insult. Documented glucose values of 3.4-3.5 mmol/L during illness before hydrocortisone supported an intermittent low-glucose tendency. HbA1c was interpreted cautiously given potential hematological confounders and was not considered independent evidence of recurrent hypoglycemia. As corticotroph failure evolved, the patient became progressively less able to tolerate ordinary physiologic insults, culminating in recurrent inflammatory crises, hemodynamic instability, electrolyte collapse, and metabolic deterioration.

This framework helps explain why the clinical phenotype repeatedly resembled sepsis. Cortisol deficiency impairs vascular responsiveness to catecholamines and angiotensin II, alters endothelial stability, and disrupts nitric oxide regulation, potentially producing vasoplegia-like distributive physiology. Simultaneously, glucocorticoid deficiency removes physiologic restraint on inflammatory cytokine signaling, including IL-1, IL-6, TNF-alpha, and interferon-mediated pathways, amplifying systemic inflammatory responses. Patients may therefore develop hypotension, inflammatory marker elevation, constitutional decline, metabolic instability, and apparent distributive shock despite absence of a persistent infectious source. In medically complex oncology patients, such physiology is particularly vulnerable to repeated misclassification as infection or chemotherapy-related deterioration [4,7,14-16].

The associated biochemical and cardiovascular abnormalities require distinction from the established adrenal insufficiency. Severe hypomagnesemia, hypokalemia, phosphate disturbances, atrial fibrillation with rapid ventricular response, and inflammatory marker elevation are not specific consequences of isolated ACTH deficiency. These abnormalities were likely multifactorial, with potential contributions from chemotherapy-associated toxicity, gastrointestinal losses, reduced nutritional intake, volume depletion, renal electrolyte handling, and pre-existing cardiovascular disease. Cortisol deficiency may nevertheless have reduced vascular responsiveness and physiologic reserve, thereby diminishing tolerance to these concurrent stressors. The proposed relationship is therefore one of impaired compensatory capacity rather than direct causation of every metabolic or cardiovascular abnormality.

The cardiovascular background substantially amplified diagnostic complexity. Mechanical mitral valve replacement, chronic anticoagulation, atrial dilation, chronic atrial fibrillation, and exclusion from anthracycline therapy because of cardiac risk rendered recurrent cardiovascular decompensation clinically plausible independent of endocrine dysfunction. Episodes of atrial fibrillation with rapid ventricular response, inflammatory surges, hypotension, and electrolyte collapse understandably directed clinical concern toward arrhythmia-mediated instability, prosthetic valve complications, or infection. However, cortisol deficiency itself may impair myocardial adrenergic responsiveness, destabilize autonomic tone, reduce vascular smooth muscle responsiveness, and lower tolerance to inflammatory and electrolyte stress. Severe recurrent hypomagnesemia and potassium fluctuations likely further amplified atrial electrical instability.

Critical illness-related corticosteroid insufficiency (CIRCI) was considered given the patient's recurrent inflammatory deterioration and ICU-level presentations. However, several features favored checkpoint inhibitor-associated ACTH deficiency. ACTH suppression was profound and reproducible, endocrine abnormalities evolved longitudinally following pembrolizumab exposure, and Synacthen testing demonstrated a pattern consistent with central adrenal insufficiency rather than transient stress physiology. Furthermore, the patient exhibited recurrent features of impaired stress adaptation, including hypoglycemia tendency, metabolic instability, electrolyte disturbances, and constitutional decline. Most importantly, glucocorticoid replacement produced rapid clinical improvement. Collectively, these findings strongly support pembrolizumab-associated isolated ACTH deficiency rather than CIRCI [14-17].

The mineral metabolism abnormalities provide an additional layer of endocrine complexity. Hypercalcemia accompanied by suppressed PTH initially raised concern for occult malignancy progression, MEN-spectrum disease, metastatic bone disease, pancreatic endocrine pathology, or calcium-parathyroid disorders. However, imaging repeatedly failed to demonstrate disseminated malignancy, pancreatic lesions, adrenal pathology, or structural endocrine neoplasia. The PTH-suppressed hypercalcemia was considered multifactorial. Adrenal insufficiency may contribute to non-PTH-mediated hypercalcemia through volume depletion and altered renal calcium handling; however, the available data do not establish cortisol deficiency as its exclusive cause. The observed biochemical abnormalities were therefore interpreted within the broader context of systemic illness, treatment exposure, and concurrent metabolic disturbances. Severe recurrent hypomagnesemia may additionally impair PTH secretion and contribute to transient calcium-phosphate dysregulation. The hypercalcemia was therefore classified as PTH-independent, with its precise mechanism remaining uncertain in the setting of malignancy, systemic illness, treatment exposure, and concurrent adrenal insufficiency.

Reduced IGF-1 and mild prolactin elevation introduce additional nuance. Although systemic inflammation, catabolic illness, nutritional compromise, and hepatic metabolic stress may suppress IGF-1, coexistence of reduced IGF-1, ACTH suppression, and mild hyperprolactinemia raises the possibility of broader but incomplete hypothalamic-pituitary involvement. Preservation of thyroid and gonadotroph axes argues against overt panhypopituitarism. The endocrine phenotype is therefore most consistent with ACTH-predominant pituitary dysfunction with possible selective or evolving involvement of additional axes, a pattern increasingly recognized in PD-1 inhibitor-associated endocrinopathy [11-14].

Exogenous glucocorticoid-induced HPA axis suppression represented an important alternative explanation and was specifically evaluated. However, the patient had received only intermittent three-day dexamethasone courses during chemotherapy, with the final exposure approximately four months before endocrine testing. She had never received chronic systemic glucocorticoid therapy. Although prolonged suppression following exogenous glucocorticoids cannot be categorically excluded, the exposure pattern and extended steroid-free interval made this explanation less likely than pembrolizumab-associated corticotroph dysfunction.

Diagnostic overshadowing was a defining feature of the patient’s clinical course. Malignancy, chemotherapy exposure, prosthetic valve physiology, recurrent atrial fibrillation, inflammatory marker elevation, anticoagulation complexity, and repeated ICU-level deterioration created multiple plausible explanations for each episode of decompensation.

Although multiple alternative diagnoses were clinically plausible, none adequately explained the full longitudinal physiologic narrative. Infection, prosthetic valve endocarditis, metastatic disease, adrenal infiltration, pancreatic pathology, MEN-spectrum disease, antiphospholipid syndrome, chemotherapy toxicity, and diabetic metabolic disease were repeatedly considered and systematically excluded through serial microbiologic evaluation, imaging, biochemical profiling, and longitudinal clinical evolution. Importantly, the diagnosis emerged not from a single decisive investigation but through longitudinal synthesis of recurrent physiologic inconsistencies across admissions.

The therapeutic response to glucocorticoid replacement provides additional biologic support for the diagnosis. Before steroid initiation, the patient experienced repeated cycles of inflammatory deterioration, hypotension, electrolyte collapse, constitutional decline, and recurrent hospitalization with ICU-level concern. Following intravenous hydrocortisone, hemodynamic stability improved, systemic symptoms lessened, metabolic deterioration stabilized, and the recurrent cycle of decompensation appeared to reverse abruptly. The temporal relationship between glucocorticoid initiation and abrupt reversal of recurrent deterioration further strengthens causal inference and supports clinically significant adrenal insufficiency [14-17].

While pembrolizumab-associated isolated ACTH deficiency is increasingly recognized, most published reports emphasize fatigue, anorexia, nausea, hyponatremia, or biochemical adrenal insufficiency rather than recurrent inflammatory and cardiovascular decompensation requiring repeated hospitalization [11-14,18-20]. The present case is distinctive because endocrine dysfunction manifested predominantly through recurrent pseudo-septic physiology, atrial fibrillation with rapid ventricular response, electrolyte collapse, MRI-negative pituitary disease, and prolonged diagnostic evolution in a patient with substantial cardio-oncology complexity.

This case highlights the importance of maintaining a high index of suspicion for checkpoint inhibitor-associated endocrinopathies in patients receiving PD-1 inhibitors. A proposed mechanism linking ACTH deficiency to the patient's recurrent multisystem decompensation is shown in Figure 3. Recurrent unexplained hypotension, inflammatory deterioration, electrolyte instability, abdominal symptoms, or apparent sepsis without convincing infectious correlation should prompt early assessment of the HPA axis, even when pituitary imaging is entirely normal.

**Figure 3:**
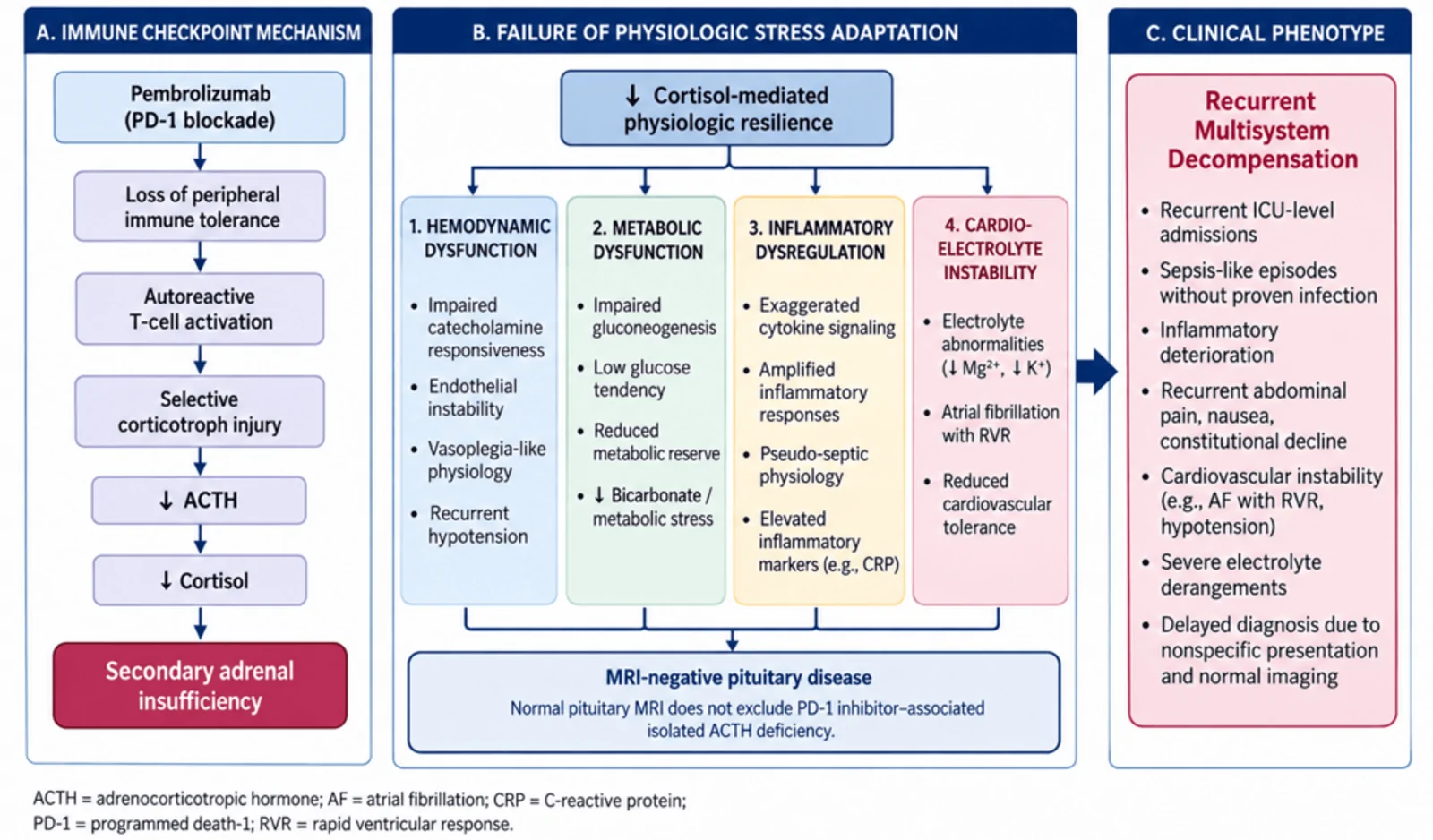
Proposed pathophysiologic mechanism of pembrolizumab-associated isolated ACTH deficiency leading to recurrent multisystem physiologic decompensation. Original schematic created by the authors using BioRender.com.

Learning points

Pembrolizumab-associated isolated ACTH deficiency may evolve gradually over several months and present through recurrent nonspecific inflammatory, cardiovascular, and metabolic deterioration rather than classic endocrine symptoms. PD-1 inhibitor-associated pituitary dysfunction may occur despite entirely normal pituitary MRI, and normal radiologic findings should not delay endocrine evaluation when biochemical or clinical suspicion exists.

Recurrent unexplained hypotension, abdominal symptoms, low glucose tendency, electrolyte instability, inflammatory deterioration, atrial fibrillation exacerbation, or recurrent sepsis-like physiology in checkpoint inhibitor-treated patients should prompt assessment of the HPA axis. Checkpoint inhibitor endocrinopathies may become diagnostically obscured in medically complex patients because symptoms overlap with infection, malignancy progression, chemotherapy toxicity, cardiovascular instability, and critical illness physiology.

## Conclusions

This case describes pembrolizumab-associated isolated ACTH deficiency presenting as recurrent multisystem physiologic deterioration in a patient with BRCA-positive TNBC undergoing neoadjuvant immunochemotherapy. Rather than manifesting through classic endocrine symptoms, adrenal insufficiency evolved gradually across multiple admissions. The case highlights several clinically important concepts. First, PD-1 inhibitor-associated endocrine toxicity may evolve insidiously over months and may remain clinically obscured despite repeated healthcare encounters. Second, clinically meaningful isolated ACTH deficiency may occur in the absence of radiologic evidence of hypophysitis, emphasizing that a normal pituitary MRI study does not exclude checkpoint inhibitor-associated pituitary dysfunction. Third, recurrent unexplained hypotension, inflammatory deterioration, low glucose tendency, electrolyte instability, constitutional decline, or apparent distributive shock in checkpoint inhibitor-treated patients should prompt early evaluation of the HPA axis, particularly when infectious, structural, and oncologic explanations remain incomplete.

The principal clinical lesson is that checkpoint inhibitor-associated central adrenal insufficiency may remain unrecognized when its manifestations are obscured by competing oncologic, cardiovascular, and inflammatory diagnoses. In this patient, severe ACTH-cortisol deficiency was demonstrated after a prolonged interval without exogenous glucocorticoid exposure, and subsequent replacement therapy was associated with sustained symptomatic recovery and absence of further comparable decompensations. Early recognition of this potentially serious but treatable endocrinopathy may prevent repeated clinical deterioration and improve continuity of care in medically complex oncology patients.
